# Shock: Assessment and management

**DOI:** 10.4102/safp.v68i1.6336

**Published:** 2026-09-30

**Authors:** Febisola I. Ajudua, Emmanuel E. Ajudua

**Affiliations:** 1 Department of Family Medicine and Rural Health, Faculty of Medicine and Health Sciences, Walter Sisulu University, Mthatha, South Africa; 2 Department of Family Medicine and Primary Health Care, Faculty of Medicine and Health Sciences, Nelson Mandela University, Gqeberha, South Africa

**Keywords:** shock, types of shock, hypovolemic shock, obstructive shock, distributive shock, cardiogenic shock, assessment of shock, management of shock

## Abstract

Shock is a clinical state where oxygen demand in the cells exceeds supply due to hypoperfusion. Early recognition of the patient with shock and a clear understanding of the possible underlying pathologies causing the shocked state aid in providing adequate interventions to mitigate the patient’s outcome and reduce the chances of morbidity and mortality. This article highlights the management of undifferentiated shock as it usually presents in primary care. It also provides information on the four main types of shock and provides a brief overview of the common underlying pathologies associated with each and highlights useful investigations that aid with early recognition and focused interventions to reverse shock.

**Contribution:**

This CPD article highlights important information for clinicians in the early identification of shock and immediate interventions in management of the shocked patient.

## Introduction

Shock can be defined as a clinical state of cellular hypoxia that results from a shortage of oxygen supply to the cells; this is often due to inadequate perfusion.^1,2^ Shock can develop from a myriad of causes, and so it is important to consider the underlying pathologies in assessing and outlining the plan of management. Shock can result in significant morbidity and mortality, especially in the resource-limited setting. Although it is not a clinical diagnosis, it is important that shock is recognised early, and correct interventions directed at the underlying pathogenic processes are initiated to prevent irreversible organ dysfunction.

## Epidemiology

A retrospective folder review conducted in a district hospital revealed that septic shock occurred in 1.9% of patients identified to have sepsis in the emergency department (ED).^3^ A more common presentation in the ED is hypovolemic shock. The other types of shock are often prevalent depending on the presence of risk for their occurrence.

## Pathophysiology of shock

In the shocked patient, hypoperfusion of tissue results in cell hypoxia; lactic acid accumulates in tissue; vasoconstriction results from the metabolic acidosis; there is a disruption of the sodium pump in the cell membrane that results in an influx of sodium and efflux of potassium, the release of intracellular toxins into the circulation and, subsequently, cell death. Figure 1 highlights the train of events and problems that result from inadequate perfusion.

**FIGURE 1:**
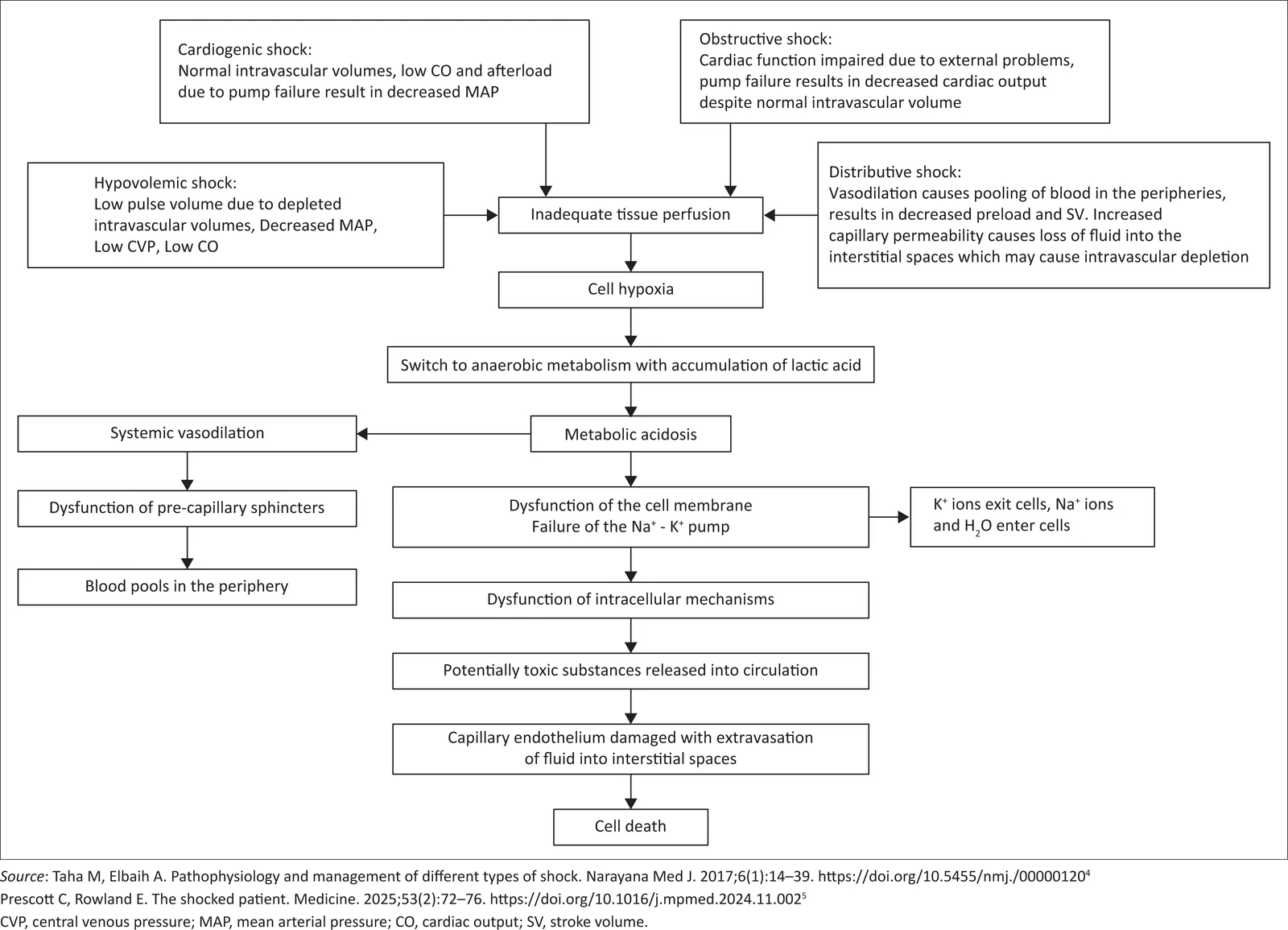
Pathophysiologic processes that arise in shock.

## Management of shock

In the initial evaluation of the shocked patient, the priority is to improve tissue perfusion and oxygenation. The format for assessing these patients is according to the Advanced Trauma Life Support (ATLS) and Advanced Clinical Life Support (ACLS) guidelines. The goal of initial management is to mitigate complications and reduce mortality.^6^

### Assessing the severity of the shocked state

The standard format for evaluating the clinical state is ‘ABCDE’. The clinician assesses the patient’s circulation using five parameters, including heart rate, blood pressure, urine output, capillary refill and the mental status. These are often used to assess the severity of shock, especially in the patient with hypovolemic shock. The pitfalls of these parameters are comorbid conditions that may interfere with readings; for instance, the hypertensive patient may have relatively normal blood pressures despite being severely shocked. No one parameter is enough; rather, a combination of all these parameters contributes to assessing the severity of shock when it is present. Table 1 highlights the classes of shock usually used in the evaluation of hypovolemic shock.

**TABLE 1:** Classes of shock.

Class	Estimated blood loss %/L	Heart rate (beats/min)	Blood pressure	Capillary refill	Respiratory rate breaths/min	Urine output (mL/h)	Mental status
1	< 15%/< 0.75L	< 100	Normal	< 2 s	14–20	30	Normal
2	15–30/0.75 L – 1.5 L	> 100	Normal to low	2–4 s	20–30	20–30	Agitated
3	30–40/1.5 L – 2 L	> 120	Low	> 4 s	30–40	5–20	Confused
4	> 40 > 2 L	> 140	Profoundly low	> 4 s	>40	Anuria	Obtunded

*Source*: Prescott C, Rowland E. The shocked patient. Medicine. 2025;53(2):72–76. https://doi.org/10.1016/j.mpmed.2024.11.002

### Types of shock

There are four main types of shock based on the pathological processes causing hypoperfusion of tissue. In assessing the patient with shock, the main concern is to reverse cellular hypoxia and tissue hypoperfusion. Table 2 highlights these four, as well as the more common aetiologies that result in shock.

**TABLE 2:** Types of shock, causes and underlying pathologies.

Type of shock	Cause	Common pathology
1. Hypovolemic shock	Blood loss	Trauma, surgery
	Dehydration	GIT losses, severe burns, third space losses
2. Distributive shock	Anaphylaxis	Histamine release, vasodilation, capillary leak
	Neurogenic	Block to the sympathetic nervous system due to lesions in the cervical or high thoracic region
	Sepsis	Vasodilation due to cytokine release, capillary leak
	Miscellaneous	SIRS, burns, pancreatitis, etc.
3. Cardiogenic shock	Acute ischaemia	Acute myocardial infarction
	Inflammatory events	Sepsis, aspiration, cardiac arrest, etc.
	Other non-ischaemic causes	Arrhythmias, valve defects, cardiomyopathies, congestive cardiac failure
4. Obstructive shock	Disorder of diastolic filling	Tension pneumothorax
		Cardiac tamponade
		Compression of the superior or inferior vena cava
	Obstruction in pulmonary circulation	Mass in heart
		Pulmonary embolism
		Amniotic fluid embolism
		Mediastinal mass compressing pulmonary vessels
	Obstruction in aortic circulation	Aortic dissection
		Leriche syndrome

*Source*: Theng L, Tan N, Dockrell L. Shock: Causes, assessment and investigation. Anaesth Intensive Care Med. 2025;27(2):78–86. https://doi.org/10.1016/j.mpaic.2025.12.005

Taha M, Elbaih A. Pathophysiology and management of different types of shock. Narayana Med J. 2017;6(1):14–39. https://doi.org/10.5455/nmj./00000120

GIT, gastrointestinal; SIRS, systemic inflammatory response syndrome.

## Investigations

### Bedside investigations

At the bedside, the clinician can assess the patient using the blood pressure (mean arterial pressure – > 65 mmHg), heart rate (> 100/min) and the shock index score (heart rate/ systolic pressure) < 0.9.^7,8^ As part of clinical examination, capillary refill time also helps with assessing tissue perfusion in the shocked patient.^9^ Other bedside tests should include a random blood glucose. When available, an arterial blood gas identifies lactate build up, metabolic acidosis that occurs due to poor tissue perfusion.^10^ The transurethral catheter to monitor urine output is a useful adjunct in the monitoring of the shocked patient; the acceptable urine output ranges between 0.5 mL/kg/h and 1.0 mL/kg/h.

### Blood tests

First-line blood tests are considered based on findings on clinical examination, and these include full blood count, renal function test, electrolyte assay and liver function tests. Also important to consider are C-reactive protein, procalcitonin and erythrocyte sedimentation rate. Other essential tests to consider, depending on the cause of shock, include type and cross-match, coagulation studies, pro-B-type natriuretic peptide assay, troponins and thyroid function tests.

### Imaging studies

These include bedside investigations:

- Electrocardiogram (ECG) – this is used to identify electrical activity in the heart; common pathologies that can be identified include abnormal heart rhythm, ischaemic changes, enlarged cardiac chambers, large pericardial effusion, cardiac tamponade, pulmonary embolism, etc.
- Point-of-care ultrasound testing – This is used for urgent assessment during the examination to identify free fluid in body cavities. Rapid ultrasound in shock protocols aid in the diagnosis of underlying problems in need of definitive care.^11^
- Echocardiogram – to assess for pericardial effusions, wall movement, valve function, etc.
- Chest X-ray, especially in patients with lung infections, effusions, etc.
- In the trauma patient, the trauma series of X-ray views is part of the initial investigations.

### Microbiologic investigations

Microbiological investigations that may be required in the first-line investigation should be focused on identifying the source of infection. In the South African setting, these would include sputum for m/c/s, tuberculosis GeneXpert (TB GXP) on relevant body fluids, blood cultures, stool investigations, urine m/c/s and other body secretions as necessary.

## Treatment

The focus of therapy in the shocked patient is to reverse tissue hypoperfusion and improve oxygenation to prevent cell death. The mnemonic for management of shock is VIP, i.e., V – ventilation, I – infuse fluids to improve perfusion of peripheral organs and P – pump. This requires the use of vasoactive substances that improve the pump pressures. Table 3 highlights the actions and end point of care in the shocked patient.

**TABLE 3:** The main focus of therapy in the shocked patient.

Intervention	Actions	End point of care
Ventilation	Airway management by ensuring patency. Provide support for the work of breathing as necessary. Determine adjuncts based on the measured oxygen saturation and other clinical signs. Relieve all impedance that may affect efficient gas exchange in the lungs. Active airway management with endotracheal intubation is required in the patient with a decreased level of consciousness, i.e., GCS < 9/15.	Patient’s airway is secure with oxygen saturation above 92%, indicating resolution of all respiratory distress.
Infusion	Ensure the patient has adequate large-bore cannulae to provide fluid therapy. Stop all active bleeding by active pressure dressings as required in the trauma patient, and use cyclocapron as required to reduce further losses. Monitor the fluid status of the patient using capillary refill time, urine output, central venous pressure and blood gas assessment. Infuse isotonic crystalloid solution starting with the bolus fluid challenge of 250 mL – 300 mL over 20–30 min to see fluid responsiveness. The preferred crystalloid in South Africa is modified Ringer’s lactate, except when contraindicated due to specific electrolyte needs. The type of fluid infused is based on the presence of active bleeding, ongoing fluid and electrolyte losses and measured levels of electrolytes and renal function. Fluid therapy should continue at a rapid pace to correct deficits, but care should be taken not to induce problems like pulmonary oedema or other stress-induced problems.	Aim for capillary refill time of < 2 s. Blood pressure > 90/60 mmHg or > 65 mmHg. Oxygen saturation > 92%. Urine output measure of > 0.5 mL/kg/h.
Pump support	In severe hypotension, it is recommended to consider the use of vasopressor support to improve systemic pressure while providing fluid therapy. The recommended vasopressor in the ED is adrenaline. It is rapid onset, short-acting and potent. It is also relatively easily available in the ED. Vasopressors are also useful in the patient with distributive shock, where the problem is pooling of fluid in the peripheries. Adrenaline is the first choice, dopamine. Inotropes are useful for support of pump failure in the patient with cardiogenic shock.	The aim is to raise the mean arterial pressure to > 65 mmHg to improve perfusion of tissue and reverse organ dysfunction.

ED, emergency department; GCS, glasgow coma scale.

Ventilation – the shocked patient should receive oxygen therapy as necessary to achieve an oxygen saturation greater than 92% for adequate tissue oxygenation. The means for oxygen delivery could vary from high-flow oxygen to endotracheal intubation with ventilatory support to ensure the reversal of all hypoxic conditions. Oxygen saturation is monitored, and arterial blood gases help to assess the partial pressures of oxygen and carbon dioxide in the blood to adjust ventilatory support as necessary.

Infuse – The initial intervention for the shocked patient is to administer bolus fluids in volumes of 250 mL – 500 mL of isotonic crystalloids such as Lactated Ringer’s solution (the preferred choice in the ED) or other balanced solutions for improving tissue perfusion.^12^ Normal saline is not a balanced solution and is not an ideal resuscitation fluid, although it is isotonic. It is considered useful in patients with severe hyponatremia or hyperkalemia requiring potassium-free fluids in resuscitation. The capillary refill time and urine output help assess the response to the fluid bolus. The pitfalls of infusing balanced fluids occur when comorbid conditions cause fluid overload, such as pulmonary oedema, peripheral oedema, cerebral oedema, etc. The clinician must recognise when the patient requires more controlled fluid infusion to avoid these complications.

Pump function – The pathophysiologic processes in the shocked patient (see Figure 1) may be a combination of different aetiologies. In some instances, vasopressors are required, especially when the patient has severe hypotension that does not immediately respond to the fluid challenge. The first-line vasopressor agent in the ED is adrenaline. It has a rapid onset of action and a short half-life. Vasopressors are particularly useful in distributive shock. In the district hospital setting, adrenaline may be the only vasopressor available. Other vasopressors available include, dopamine and phenylephrine. In cardiogenic shock, it might be necessary to consider the use of inotropes; these patients are commonly managed in higher levels of care.

Other considerations in the management of shock often rely on the underlying causes of shock and any other pathologic processes, such as sepsis, etc.

## Conclusion

Although there have been many advances in therapy in the last several decades, there is still a high risk for mortality and irreversible morbidity linked with shock, especially in resource-constrained settings. Several patient factors influence the prognosis in these patients. It remains necessary that all frontline clinicians are skilled in basic principles for the early recognition and management of patients presenting in shocked states.
